# Supplementary material for: A Qualitative Exploration of Fijian Perceptions of Diabetes: Identifying Opportunities for Prevention and Management
Source: Int J Environ Res Public Health. 2019 Mar 27;16(7):1100. doi: 10.3390/ijerph16071100 (PMC6480118; doi:10.3390/ijerph16071100)
Supplement: Supplementary file 1 [file ijerph-16-01100-s001.pdf]

Interview schedule: Diabetes prevention among Fijians

Note for interviewer:

- Main questions are provided beside filled dot points.
  - Optional prompt questions are provided beside unfilled dot points if required

1. Introduction

- How often do you visit Fiji?
  - How much contact do you have with family and friends in Fiji?
  - How does that influence your life in Australia?
- Please list for me the common health conditions that impact Fijian people?
  - Is there any difference that you are aware of between Fijians living in Australia or those living in Fiji?
- If you don't mind sharing, do you know anyone who has diabetes?

2. Perceptions of diabetes

- Tell me about what you know about diabetes; what it means to you?
  - If you worry about diabetes, tell me about how big a problem you think the condition is and why?
- How big an issue do you feel diabetes is, in your community?

3. Knowledge of diabetes

- How would you explain what diabetes is to someone that did not know anything about it?
  - What do you think are the causes of diabetes?
  - How do you think diabetes can be controlled/managed?
  - What do you think are the health consequences of having diabetes?

4. Facilitators and barriers to preventing/managing diabetes

- Can you think of issues/activities in daily Fijian culture that can promote early development of diabetes, please give as many examples that you can
- If a person did develop diabetes, what may stop them from keeping it under control and why?

5. Ideas for support

- In Fiji, the church and Chiefs provide leadership in the community, particularly on the islands and in villages. Is there any parallel influence in Australia?
  - Where do Fijians meet together in Australia?

- You are the newly appointed high chief and some funding from the local government has been given to you. How would you use your skills and position to help prevent diabetes?
- What could be done within your community to help prevent diabetes developing in individuals?
  - What would be involved?
  - Who would be involved?
  - What would they do?

- How would they do this?

- What could be done within your community to help people with diabetes look after their condition better?
  - What would be involved?
  - Who would be involved?
  - What would they do?
  - How would they do this?

6. Physical activity participation

- Describe what sort of physical activities (exercise), Fijians participate in – in Australia? Does it differ in Fiji?
  - High school aged
  - 20's
  - 30's

- What can be done to help Fijians in your community who are in their 30s and onwards to participate in these physical activities?
  - What are the barriers to participation that need to be overcome?
  - What are the facilitators to participation that could be focused on to encourage further participation?

7. Dietary intake

- Describe the typical diet of Fijians in Australia
- As a community leader what can be done to help Fijians in your community to make healthier food choices? What resources would you need?
- How does the diet of Fijians in Australia differ from family living in Fiji?

8. Body Image Perception

- Using prompt cards showing body shapes
  - Which female/male body figure is most healthy
  - Which female/male body figure is most attractive
- How has this changed since coming to Australia

9. Readiness for change

- Do you think many Fijians need to, or want to, lose weight? Explain your thinking

10. Other

- Any further comments about anything that has been discussed?
